# Processionary caterpillar reactions in Southern Italy forestry workers: description of three cases

**DOI:** 10.1186/s12948-021-00155-8

**Published:** 2021-09-06

**Authors:** Luisa Ricciardi, Concetto Giorgianni, Giusi Briguglio, Sebastiano Gangemi, Giovanna Spatari

**Affiliations:** 1 School and Unit of Allergy and Clinical Immunology, Department of Clinical and Experimental Medicine, University of Messina, AOU Policlinico “G.Martino”, Via Consolare Valeria 1, 98124 Messina, Italy; 2 Department of Biomedical Sciences, Dental, Morphological and Functional Investigations, University of Messina, Via Consolare Valeria 1, 98124 Messina, Italy

**Keywords:** Processionary caterpillar, Forestry workers, Skin reactions, Respiratory reactions, Contact reactions, Airborne reactions

## Abstract

**Background:**

Processionary caterpillar (PC), also named *Thaumatopea pityocampa*, has been reported to cause hypersensitivity reactions after contact with a toxin contained in hair-like bristles which cover this insect. Occupational exposure to PC is underestimated in outdoor workers and especially in forestry workers (FW) and is globally diffusing because of rising temperatures.

**Cases presentation:**

We present the first three cases of FW from Sicily, a Southern Italy (SI) region, which reported hypersensitivity reactions due to exposure to PC infested trees. These cases were identified by the occupational health physician during the annual screening of FW working in the Mountains of north-eastern Sicily. Interviewing a population of 630 FW, 1 male and 2 females reported direct contact skin reactions together with airborne contact reactions to PC hairs causing mild respiratory symptoms in two cases and ocular symptoms in one case, which needed treatment with systemic corticosteroids and antihistamines.

**Conclusions:**

This is the first report of hypersensitivity reactions in SI FW due to occupational exposure to PC. Further screenings not only in FW but also in other populations of outdoor workers are needed in order to assess the real incidence of contact and airborne reactions due to occupational exposure to PC. Though so far no correlation has been found with atopy, it seems apparent that the reactions occur in susceptible subjects; further research is needed for a correct diagnosis and to identify possible desensitization procedures.

## Background

**Fig. 1:**
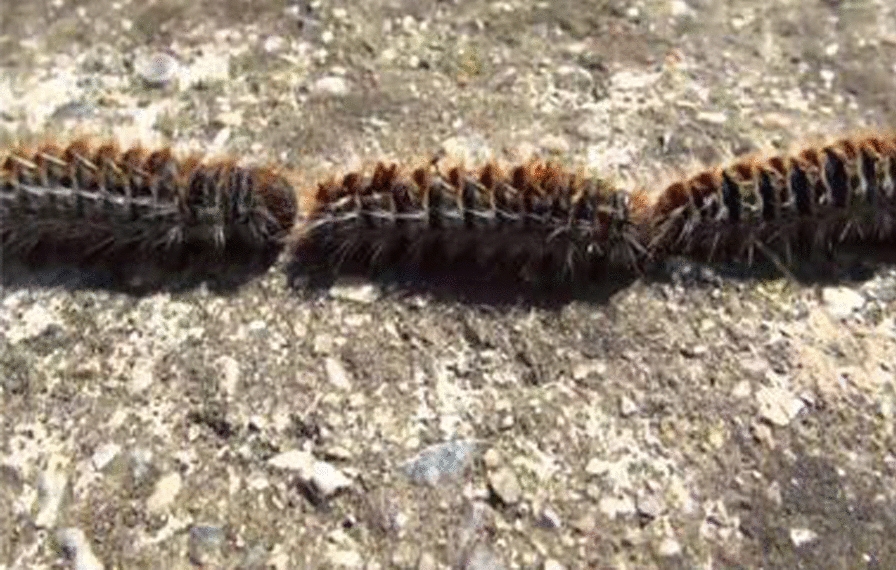
Caterpillars moving as in a procession and so called processionary

Exposure to processionary caterpillar (PC) has been reported to cause both local and systemic reactions [1]. PC is an insect of the Lepidoptera order which owes its name to the strange way they have to move all together in line as if they were in a procession (Fig. 1).

**Fig. 2:**
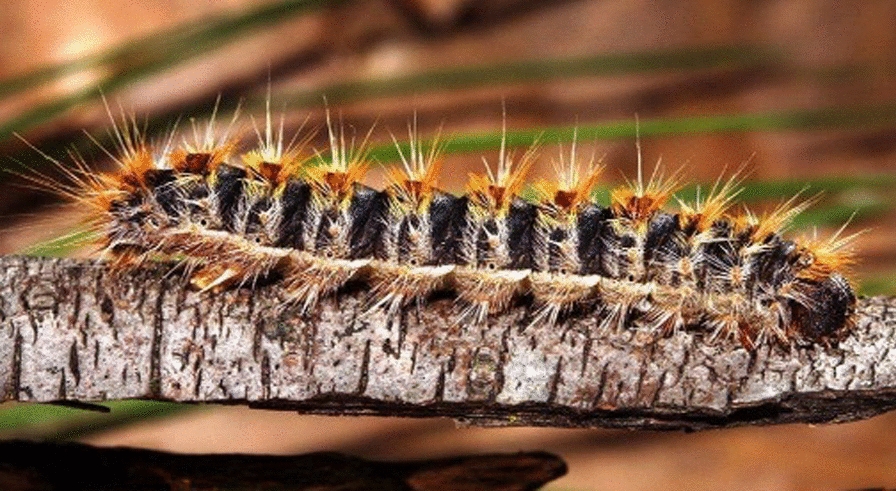
Processionary caterpillar covered with hair-like bristles called setae responsible for reactions

These insects are also known with their original latin name *Thaumatopea pityocampa*; they include butterflies and moths undergoing complete metamorphosis. The larvae commonly called caterpillars are covered in hair-like bristles called setae (Fig. 2) containing an urticating toxin named thaumetopoein [2].

**Fig. 3:**
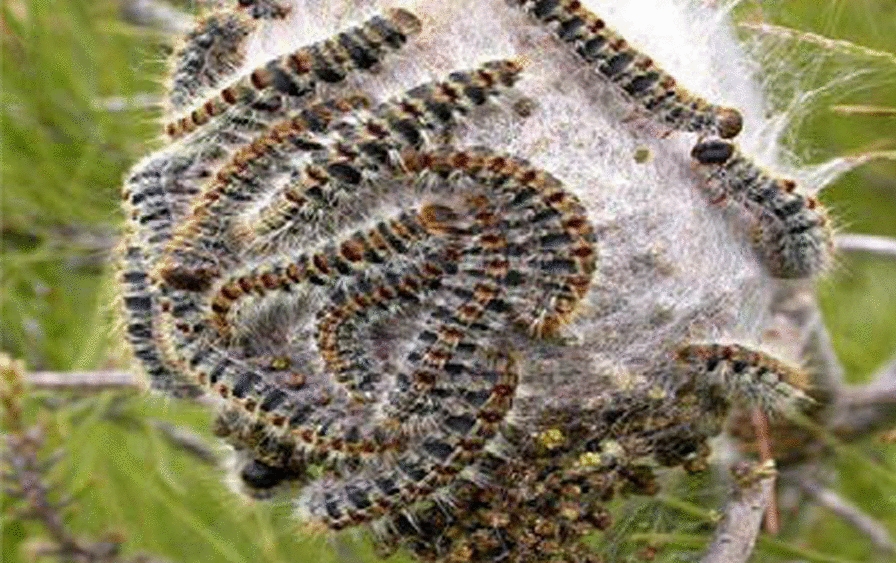
Processionary caterpillars’ nest covered with larvae

Skin, eyes and upper airways can be affected by direct or airborne contact with PC hairs from nests or caterpillars (Fig. 3).

Air dispersed PC hairs have been reported to cause contact dermatitis or aero-mediated contact dermatitis, itching rash, allergic conjunctivitis, dyspnea and wheezing Furthermore, systemic reactions other than urticaria have been reported such as anaphylactic shock [3, 4].

Up to now there are no sufficient data on the incidence of reactions induced by processionary moth in the general population even if it seems that it is an underestimated problem [5]. Health concerns correlated to the urticating setae are secondary to the PC population extension depending on climate warming [6].

**Fig. 4:**
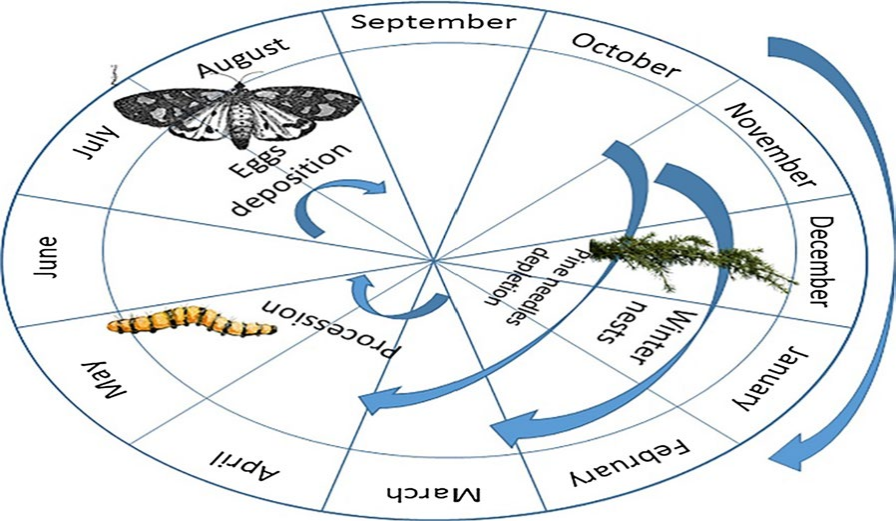
Processionary caterpillar life cycle

PC has a life cycle which follows different steps in different periods of the year (Fig. 4). In autumn eggs remain on tree branches covered with greyish scales while pine needles deplete; in spring larvae hatch from eggs and caterpillars gradually grow in length covered by setae; in summer, from mid July, moth start emerging and deposit eggs.

**Fig. 5:**
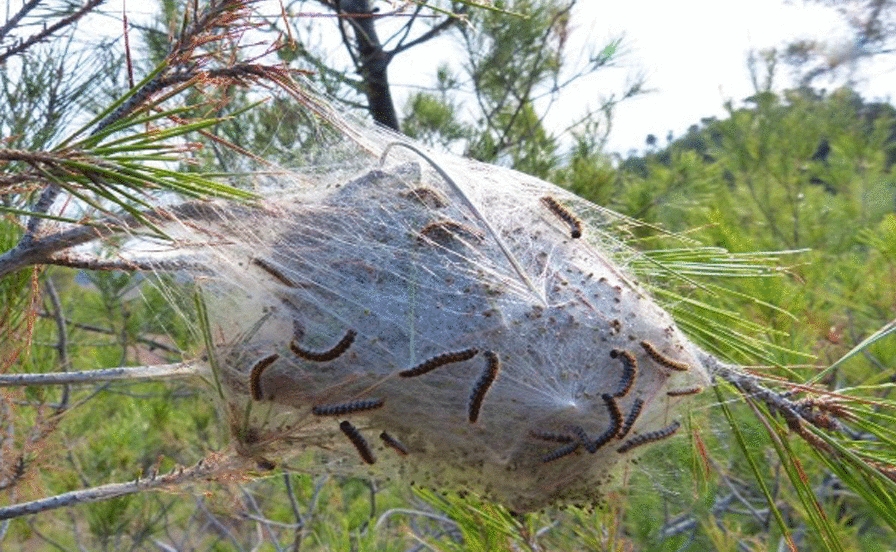
Processionary caterpillars often nest on pine tree branches which are their usual natural environment

Occupational exposure to PC has been reported even if in forestry workers (FW) it is underestimated. These insects usually are present in pine and oak woods were nests can be seen through the branches (Fig. 5).

A survey on a FW population in northern Italy (NI FW) has been previously carried out [7] but no data have been ever reported on southern Italy FW (SI FW).

## Case series

During the annual scheduled examination of a population of 630 FW in the Mountains of north-eastern Sicily, the occupational health physician asked the workers to report if they had ever experienced a reaction after exposure to PC.

Among all interviewed FW, 1 male and 2 females, reported at least one reaction after exposure to trees infested by PC, either pines or oaks.

The first case was a 38-year-old female who had been working for 18 years. She reported two episodes of airborne urticaria without any direct contact with PC. The first episode had occurred about 10 years before with intense itching and rashes in both upper limbs which recovered after using antihistamine topical treatment. After avoiding working in pine woods she, nonetheless, experienced a second episode because of the presence of PC in other conifer trees, the Nebrod Fir, also known as the Sicilian Fir or Abies Nebrodensis in the Nebrodi Mountains of north-eastern Sicily. In this occasion she experienced not only skin reactions but also mild respiratory symptoms such as cough and dyspnea which resolved after oral corticosteroid treatment.

The second case was a 44-year-old male who had been working for 24 years. He referred to be allergic to Parietaria pollens and had experienced contact urticaria when touching nests and caterpillars not only on pines but also on oak trees. He also complained of conjunctivitis which was worse than seasonal spring allergic conjunctivitis from exposure to Parietaria pollens’ peaks. Oral clorpheniramine intramuscular treatment followed by a 7-days course of oral antihistamines was needed.

The third case was a 52-year-old female who had been working for 31 years. She reported to have experienced itchy wheals and rashes especially at her neck as well as transitory dyspnea which were treated with oral corticosteroids and oral antihistamines.

## Discussion and conclusions

PC is very common in pine woods so it is also named as Pine Processionary Moth of the Mediterranean area even if global warming on the other hand is increasing the spread of these insects with the possible occurrence of reactions also in other areas [8]. Furthermore, not only pines can be infested by processionary caterpillar but also other trees such as oaks in further northern areas than the Mediterranean [9].

Occupational reactions in FW are mostly correlated to Hymenoptera stings [10] and tick transmitted infections [11]; among other occupational allergens, PC larvae and their hairs suspended in the air, have been reported to affect not only FW but also other occupational categories of wood collectors and cutters, farmers, stockbreeders, construction workers, residential gardeners and entomologists [12]. As reactions do not occur in all exposed FW it is more likely that they occur in susceptible subjects. Up to now no correlation has been found either with atopy, even if one of our female FW was allergic to Parietaria pollens causing conjunctivitis, or gender. In general males have a higher prevalence of self-reported symptoms even if these data do not correlate to our survey results as in our population two women and only one man reported hypersensitivity reactions to processionary caterpillar. Occupational exposure in general and in particular correlated to frequency of visits in woods and daily exposure are considered as risk factors for reactions to PC [13]. FW screened in our survey had been working for several years but only as seasonal workers; this may correlate to the fewer number of FW who presented hypersensitivity reactions in 0.4% of SI FW (3/630) rather than 26.3% NI FW (24/91) as previously reported [6].

The intensity of the reaction to PC in FW could also be influenced by exercise as FW do an intense physical work; exercise-induced anaphylaxis (EIA), including skin and respiratory symptoms as described in the above reported cases, can occur after physical activity, influenced by cofactors including high temperatures [14].

Reactions due to processionary caterpillar exposure have been reported to have an IgE-mediated pathogenesis even if further studies are still needed; skin prick tests and specific IgE with setae and whole larval extracts have been performed with a low percentage of positive response [15]. Extracts with high specificity and sensibility are still not yet commercially available on a large scale but two major allergens Tha p 1 and Tha p 2, which have shown no homologies to other insects, have been isolated [16, 17].

Further investigation is needed in order to determine possible genetic predisposing factors or underlying systemic diseases causing reactions, even other than occupational, after exposure to PC.

## Data Availability

The dataset used in this study is available with authors and can be made available upon reasonable request.

## Abbreviations

PC: Processionary caterpillar
FW: Forestry workers
NI: Northern Italy
SI: Southern Italy
